# Impaired cecal motility and secretion alongside expansion of gut-associated lymphoid tissue in the *Nlgn3*^*R451C*^ mouse model of autism

**DOI:** 10.1038/s41598-023-39555-y

**Published:** 2023-08-04

**Authors:** Chalystha Yie Qin Lee, Gayathri K. Balasuriya, Madushani Herath, Ashley E. Franks, Elisa L. Hill-Yardin

**Affiliations:** 1 School of Health and Biomedical Sciences, RMIT University, 223, Bundoora West Campus, 225-245 Clements Drive, Bundoora, VIC 3083 Australia; 2 Graduate School of Medicine, Kobe University, Kobe, Japan; 3 Department of Pathology and Immunology, Baylor College of Medicine, Houston, TX USA; 4 Department of Pathology, Texas Children’s Microbiome Center, Texas Children’s Hospital, Houston, TX USA; 5 Department of Physiology, University of Melbourne, Parkville, VIC Australia; 6 School of Life Sciences, La Trobe University, Bundoora, VIC Australia

**Keywords:** Immunology, Neuroscience, Diseases, Gastroenterology, Medical research

## Abstract

Individuals with Autism Spectrum Disorder (ASD; autism) commonly present with gastrointestinal (GI) illness in addition to core diagnostic behavioural traits. The appendix, or cecum in mice, is important for GI homeostasis via its function as a key site for fermentation and a microbial reservoir. Even so, the role of the appendix and cecum in autism-associated GI symptoms remains uninvestigated. Here, we studied mice with an autism-associated missense mutation in the post-synaptic protein neuroligin-3 (*Nlgn3*^*R451C*^), which impacts brain and enteric neuronal activity. We assessed for changes in cecal motility using a tri-cannulation video-imaging approach in ex vivo preparations from wild-type and *Nlgn3*^*R451C*^ mice. We investigated cecal permeability and neurally-evoked secretion in wild-type and *Nlgn3*^*R451C*^ tissues using an Ussing chamber set-up. The number of cecal patches in fresh tissue samples were assessed and key immune populations including gut macrophages and dendritic cells were visualised using immunofluorescence. *Nlgn3*^*R451C*^ mice displayed accelerated cecal motor complexes and reduced cecal weight in comparison to wildtype littermates. *Nlgn3*^*R451C*^ mice also demonstrated reduced neurally-evoked cecal secretion in response to the nicotinic acetylcholine receptor agonist 1,1-dimethyl-4-phenylpiperazinium (DMPP), but permeability was unchanged. We observed an increase in the number of cecal patches in *Nlgn3*^*R451C*^ mice, however the cellular morphologies of key immune populations studied were not significantly altered. We show that the R451C nervous system mutation leads to cecal dysmotility, impaired secretion, and neuro-immune alterations. Together, these results suggest that the R451C mutation disrupts the gut-brain axis with GI dysfunction in autism.

## Introduction

The human appendix functions as a reservoir for commensal bacteria^1,2^, rather than a vestigial remnant of the human digestive tract^3,4^. In rodents, the cecum is synonymous with the human appendix, located at the junction of the distal ileum and proximal colon. It is enriched with mucus biofilms that support the growth and maintenance of gut flora. This environment allows for the re-inoculation of the colon when purged following diarrhoea or illness^1^. Functions of the cecum also include fermentative digestion, short-chain fatty acid (SCFA) production^5^ and immune regulation via resident gut-associated lymphoid tissue (GALT), termed the cecal patch. The cecal patch regulates immunoglobulin A secretion that modulates immune selectivity^6,7^. Similarly, in humans, the appendix is rich in lymphoid tissue essential to antibody production and T and B lymphocyte maturation^4,6,8^. Together with the enteric nervous system (ENS), situated in close proximity to the GALT and cecal microbial reservoir, these three components carry out essential neuro-immune crosstalk for GI homeostasis^9^.

Comprising the myenteric and submucosal plexus, the ENS primarily functions to regulate gut motility and permeability, respectively. Yet, our understanding of cecal motility and intestinal barrier function, which altogether mediate cecal immune and microbial functions in mammals in gastrointestinal (GI) health and disease, is less well-developed. Notably, in cases of childhood neurological conditions such as Autism Spectrum Disorder (ASD; autism), characterised by cognitive, behavioural, and/or physical impairments arising from disruptions in neurodevelopment^10^, GI illness is documented 4 to 8 times more frequently compared to neurotypical children^11–15^. Indeed, higher incidences of constipation, diarrhoea, and stomach discomfort are reported in children with autism. Such GI symptoms are furthermore associated with autism severity, social disengagement, irritability and anxiety^16^. Of note, levels of inflammatory cytokines are higher in children with autism and correlate with symptom severity^17,18^, and these changes are further correlated to key autism-associated bacterial populations (e.g., Clostridiales) and GI symptoms^19^. Critically, such features reported in humans are documented in preclinical mouse models, altogether highlighting neuro-immune dysfunction as central pathological features in autism-relevant endophenotypes in mammals^20^.

Mice harbouring an *Nlgn3*^*R451C*^ mutation represent a well-established mouse model of autism. Genetically engineered to express an Arg to Cys substitution at position 451 of the human Neuroligin-3 polypeptide sequence^21–32^, this mutation was first reported in two Swedish brothers^33^ diagnosed with autism and prominent GI symptoms including oesophageal regurgitation, gastrointestinal inflammation and diarrhoea^34^. Upon closer evaluation of the brothers’ clinical records (Supplementary Note S1 in Hosie et al. 2019), we noted significant reoccurrence of gastrointestinal pain symptoms including “chronic abdominal pain”, “gastrointestinal upset”, and “chronic intestinal pain”. Several studies have linked abdominal pain with prolonged bloating and gastrointestinal gas retention or transit due to inefficient removal of gas^35–37^. Gastrointestinal gas, alongside SCFA, are by-products of carbohydrate fermentation, a process which occurs primarily in the appendix (humans) and cecum (rodents)^5^. It is therefore vital to comprehensively characterize the physiology of the appendix/cecum in this pre-clinical mouse model to identify converging mechanisms exerted by a point mutation, such as *Nlgn3*^*R451C*^, that is associated with autism and GI dysfunction.

Notably, studies of *Nlgn3*^*R451C*^ mice show GI changes including accelerated small intestinal transit in vivo and hypersensitivity to GABA_A_ receptor modulation ex vivo^34^. Furthermore, populations of enteric neurons that express nitric oxide synthase (NOS), a key gut neurotransmitter, in the small intestine and cecum of these mice are also altered^34,38^, suggesting that such a loss could drive GI dysfunction. Furthermore, we previously reported decreased cecal weight and changes in constituent immune cells consistent with aberrant neuro-immune interactions in *Nlgn3*^*R451C*^ mice, including altered density and morphology of cecal-patch macrophages^38^. Here, we closely examine cecal function including digestive churning, immune function, and intestinal barrier integrity in *Nlgn3*^*R451C*^ mice to understand the impact of such a mutation on GI function in autism.

## Results

### Distinct neurogenic contraction pattern observed in the cecum: Cecal Motor Complex (CeMC)

Cecal motor complexes (CeMCs) are clusters of individual contractions that typically begin with a forward contraction travelling from cecal tip to colon, followed by multiple reverse and forward contractions. In studies of rabbit cecum motility^39^, these are referred to as pro and anti-peristaltic contractions, respectively. In mice, we observed that these clusters or complexes are interspersed by periods of quiescence where little to no contraction activity occurs.

**Figure 1:**
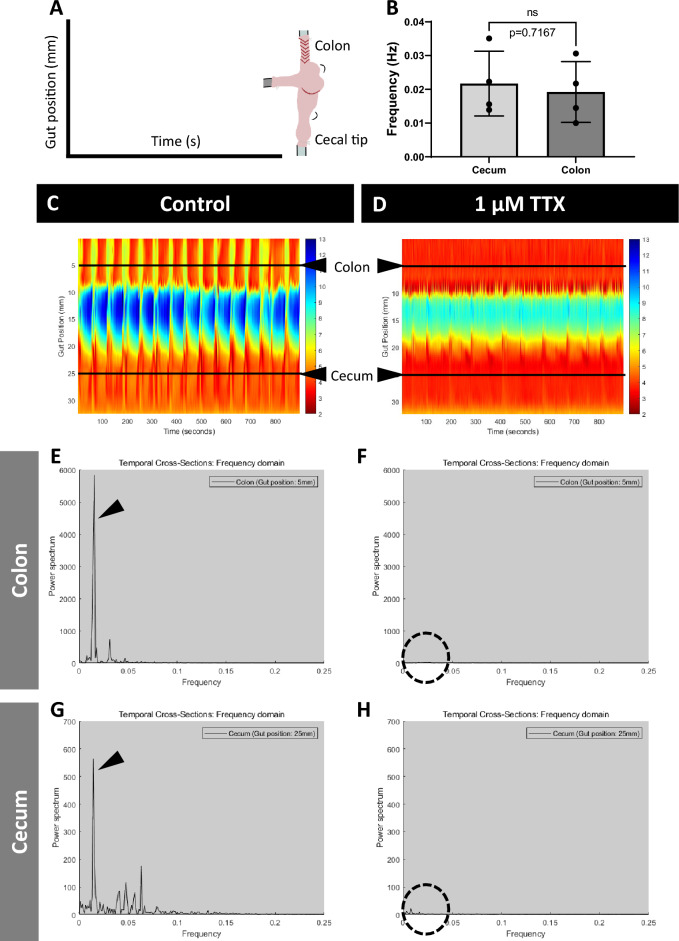
Novel neurogenic contraction patterns are observed in the cecum -Cecal Motor Complex (CeMC). (**A**) Representative diagram to show how the cecal preparation in the organ bath is mapped onto the spatiotemporal heatmaps ((**C**) and (**D**)). The x-axis refers to time in seconds, whereas the y-axis refers to gut position (mm). (**B**) Fast Fourier transform shows that the dominant oscillation of the contractile pattern in both the cecum and colon occur at a similar low frequency (arrowheads on (**E**) and (**G**)). Statistical comparisons between cecum and colon were conducted using Student’s unpaired *t*-test. Individual data and mean ± SD were plotted for n = 4 mice in each group. ns = p > 0.05 (**C**) Representative spatiotemporal heatmap of the contractile activity observed in the cecum and connecting proximal colon, at control. (**D**) Representative spatiotemporal heatmap showing that the majority of the contractile activity is abolished in both the cecum and colon in the presence of 1 μM TTX in the organ bath. Power spectrum graphs in the colon at (**E**) control and (**F**) TTX show that contractile activity is entirely abolished with TTX, as expected (dotted circle). In the cecum, the power spectrum graphs at (**G**) control show a similar trace to that found in the proximal colon (**E**). (**H**) In the presence of TTX, the majority of contractile activity in the cecum is similarly abolished (dotted circle). Heatmaps and power spectrum graphs shown are representative of preparations from n = 4 WT mice. WT= wildtype.

In fresh ex vivo cecal preparations, we observed clustered, rhythmic contractile patterns with a regular periodicity when observed in real time (Supp. Video 1). To investigate whether these cecal contractile patterns were neurogenic or myogenic in nature, we added tetrodotoxin (TTX) to a subgroup of experiments and assessed for changes in motility. TTX is a Na_v_ channel blocker that blocks tonic inhibition in the ENS, resulting in the abolishment of colonic migrating motor complexes (CMMCs) and increased muscle tone in the colon (reviewed by Smith and Koh^40^ and Spencer et al.^41^). We also aimed to compare cecal motility with well-established motility patterns in the colon. Consistent with previous studies on colon motility, we report that contractions in the colon segment adjacent to the cecum in our preparations are abolished with TTX (Fig. 1C–F). We note that due to physical limitations, we were only able to study TTX responses in the proximal colon and could not determine whether these contractions can be classified as CMMCs. Fast Fourier transform analyses showed that the dominant oscillation of the contractile pattern in the colon occurs at a low frequency of 0.019 ± 0.008 Hz (Fig. 1E). This low-frequency peak was abolished by addition of TTX (Fig. 1F) suggesting that this component is likely neurogenic. Overall, we observed cecal contractile activity in spatiotemporal heatmaps and power spectrum graphs, similar to that found in the colon. Fast Fourier transform analysis revealed that low-frequency oscillations in the caecum had a frequency of 0.022 ± 0.009 Hz (Fig. 1G). In the presence of TTX, the majority of cecal contractile activity is abolished (Fig. 1H). When comparing dominant low-frequency oscillations in cecum and colon, there is no significant difference between the two gastrointestinal regions (Fig. 1B). Our results suggest that contraction patterns in the cecum are neurogenic, resembling CMMCs in the colon.

**Figure 2:**
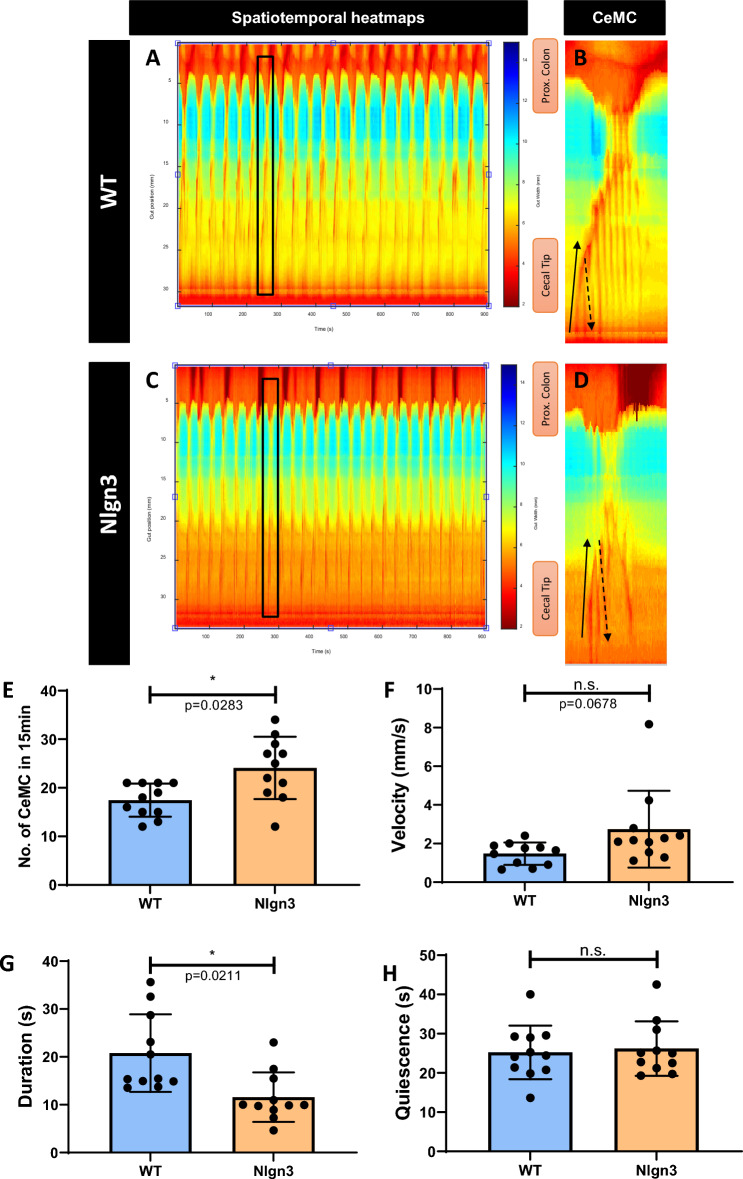
Cecal motility patterns are altered in *Nlgn3*^*R451C*^ mice. (**A–D**) Representative spatiotemporal heatmaps of cecal motor complexes (CeMC) (denoted by black boxes and enlarged in (**B,D**) in (**A-B**) wildtype (WT) and (**C,D**) Nlgn3 mice. Forward contractions (FC) are denoted by black arrows and reverse contractions (RC) are denoted by dashed arrows. n = 11 in each group. (**E**) Nlgn3 mice have a higher number of CeMCin 15 min. (**F**) Velocity of CeMC between WT and Nlgn3 is not significantly different. (**G**) Duration of CeMC in Nlgn3 are shorter compared to WT. (**H**) No difference in quiescence period in WT and Nlgn3 mice. CeMC frequency was analysed using a Mann–Whitney statistical test. Other parameters of cecal motility were analysed using Student’s unpaired *t-*test. All measures of cecal motility were assessed using the Benjamini–Hochberg procedure to account for false discovery rate. Individual data and mean ± SD were plotted for n = 10–11 mice in each group. *n.s.* not significant. ns = p > 0.05, *p < 0.05. WT = wildtype; Nlgn3 = *Nlgn3*^*R451C*^.

Upon closer investigation of individual complexes, each cluster of contractions comprises multiple forward (from cecal tip to colon) and reverse (from colon to cecal tip) contractions, which we collectively referred to as Cecal Motor Complexes (CeMCs) (Fig. 2A–D).

### Impaired cecal motility in *Nlgn3*^*R451C*^ mice

CeMCs in mutant tissue appear less complex than those observed in wildtype (WT) mice, with fewer forward and reverse patterns within each complex (Fig. 2A,B wildtype, 2C-D Nlgn3). CeMC frequency was higher in *Nlgn3*^*R451C*^ mice (Fig. 2E) and there was a trend for an increased in the speed of propagation in *Nlgn3*^*R451C*^ cecum samples (Fig. 2F). CeMCs were also of shorter duration in *Nlgn3*^*R451C*^ cecum compared to WT (Fig. 2G), while inter-CeMC quiescence intervals were not significantly different (Fig. 2H).

**Figure 3:**
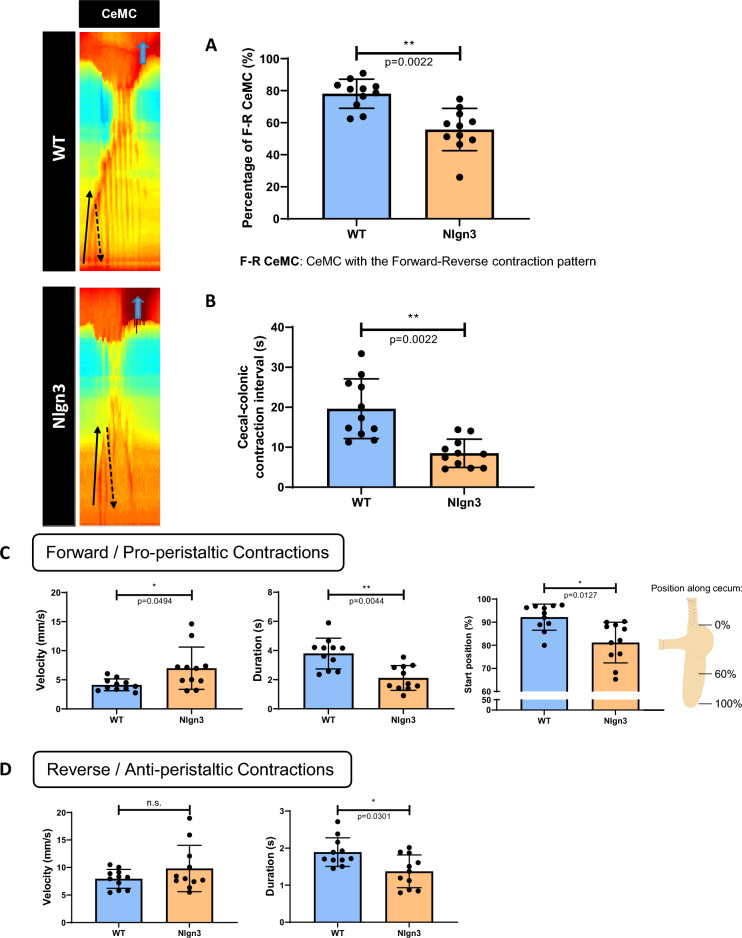
Altered cecal contraction patterns in *Nlgn3*^*R451C*^ mice. (**A**) The percentage of CeMCs showing the contraction pattern is significantly lower in Nlgn3 mice. (**B**) The cecal-colonic contraction interval is significantly shorter in Nlgn3 mice. (**C,D**) Characteristics of individual contractions within CeMCs. (**C**) Alterations to velocity, duration, and start position of the forward contraction (FC) in Nlgn3 mice. (**D**) For reverse contractions (RC), the velocity is comparable, however their duration is shorter in Nlgn3 mice compared to WT. Cecal motility parameters were analysed using Student’s unpaired *t-*test. All measures of cecal motility were assessed using the Benjamini–Hochberg procedure to account for false discovery rate. Individual data and mean ± SD were plotted for n = 11 mice in each group. ns = p > 0.05, *p < 0.05, **p < 0.01. WT = wildtype; Nlgn3 = *Nlgn3*^*R451C*^.

We observed a canonical contraction pattern within an individual CeMC, where the forward contraction (FC) is followed by one or more reverse contractions (RC). Notably, the percentage of CeMCs with this contraction pattern is reduced in *Nlgn3*^*R451C*^ mice compared to WT (Fig. 3A) suggesting cecal dysmotility in the mutant mice. Interestingly, in both genotype groups, these cecal motility patterns appear to correlate with or trigger contractile activity in the attached proximal region of the colon. The cecal-colonic contraction interval (i.e., the time elapsed between the start of the FC of the CeMC and the start of a colonic contraction), was altered in mutant mice. In CeMCs that precede a colonic contraction, the interval between these two motor patterns was shorter in *Nlgn3*^*R451C*^ mice compared to WT (Fig. 3B). This finding could suggest that altered cecal motility impacts downstream colonic dysmotility.

We further investigated CeMCs at higher image resolution to examine individual forward (FC) and reverse (RC) contractions within contraction clusters. For each video recording, 3 CeMCs were selected for analysis. The first observed FC of these 3 CeMCs was examined. For analysis of reverse contractions, the first RC after the initial FC of these 3 CeMCs was also assessed. In *Nlgn3*^*R451C*^ mice, FCs had a higher propagation velocity and shorter duration and were initiated more distally (i.e., closer to the colon) compared to WT. In contrast, FCs in WT mice originated from the cecal tip prior to travelling distally towards the colon (Fig. 3C). The propagation velocities of RCs in *Nlgn3*^*R451C*^ mice were similar to WT, however these were shorter in duration (Fig. 3D). CeMC measurements are presented in Supp. Table 2.

### Cecal content is reduced in *Nlgn3*^*R451C*^ mice

**Figure 4:**
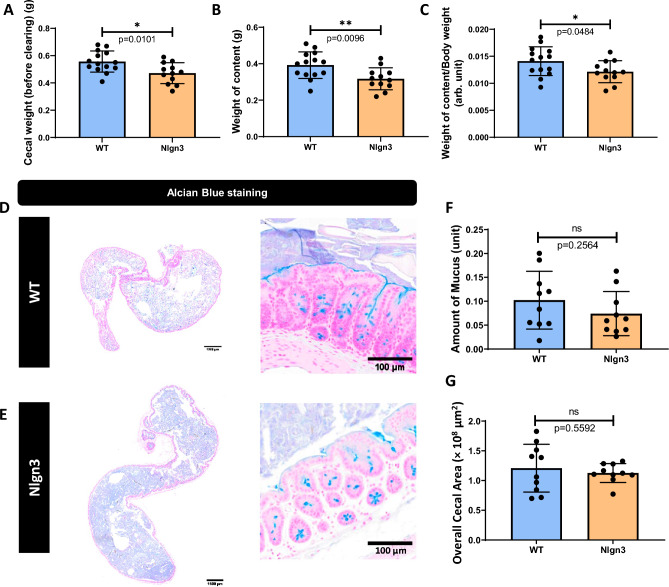
Cecal content is reduced in *Nlgn3*^*R451C*^ mice. (**A**) Cecal weight is decreased in Nlgn3 mice. (**B**) The weight of cecal content is reduced in Nlgn3 mice compared to WT. (**C**) The weight of cecal content standardized to body weight is significantly lower in Nlgn3 mice. Statistical comparisons were conducted using Student’s unpaired *t*-test and individual data and mean ± SD were plotted for WT, n = 14; Nlgn3, n = 12 mice in each group. *p < 0.05, **p < 0.01. Mucus content and overall cecal area in WT versus Nlgn3 mice. Cecum cross-sections stained with Alcian Blue from (**D**) WT and (**E**) Nlgn3 mice. (**F**) There is no significant difference in amount of stained mucus in cecal cross-sections between WT and Nlgn3 mice. (**G**) There is no significant difference in overall cecal area between WT and Nlgn3 mice. Statistical comparisons were conducted using Student’s unpaired *t*-test. Individual data and mean ± SD were plotted for n = 10 mice in each group. ns = p > 0.05, *p < 0.05, **p < 0.01. WT = wildtype; Nlgn3 = *Nlgn3*^*R451C*^.

Because decreased cecal weight was previously reported in *Nlgn3*^*R451C*^ and Nlgn3^−/−^ knockout mice^38^, we investigated whether mucus and overall cecal content were potential contributors to cecal weight. As expected, total cecal weight was significantly lower in *Nlgn3*^*R451C*^ mice compared to WT littermates (Fig. 4A). Cecal content weight was also reduced in *Nlgn3*^*R451C*^ compared to WT samples (Fig. 4B). When normalized against body weight, cecal content in *Nlgn3*^*R451C*^ mice was also significantly lower than in WT littermates (Fig. 4C). Yet, cecal cross sections from wildtype and *Nlgn3*^*R451C*^ mice showed comparatively similar visible mucus content (Fig. 4D–F). Despite the reduction in weight, cecal cross sectional area was similar in wildtype and *Nlgn3*^*R451C*^ mice (Fig. 4G). Cecal weight and mucus content data are presented in Supp. Table 3.

### Decreased neurally-evoked secretion in the *Nlgn3*^*R451C*^ cecum

**Figure 5:**
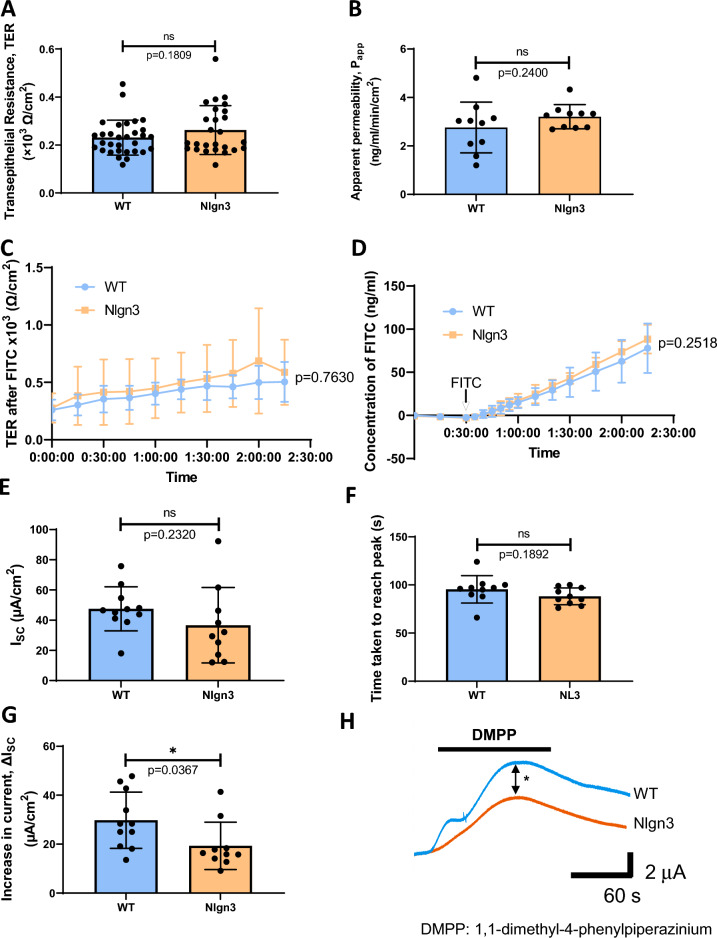
Transepithelial resistance (TER) and paracellular permeability of WT and *Nlgn3*^*R451C*^ mice. There is no significant difference observed in the TER (**A**) and apparent permeability (**B**) of WT and Nlgn3 cecum. The TER over time (**C**) as well as concentration of FITC-Dextran (**D**) was not significantly different in WT and Nlgn3 caeca. Statistical comparisons were conducted using (**A,B**) Student’s unpaired *t-*test and (**C,D**) two-way ANOVA with Šídák multiple comparisons test. Individual data and mean ± SD were plotted for (**A**) WT: n = 31; Nlgn3: n = 26 and (**B–D**) n = 10 in each group. ns = p > 0.05. Short-circuit current, I_SC_, response to 20 μM DMPP. (**E**) The I_SC_ at baseline is not significantly different in Nlgn3 compared to WT. (**F**) Time taken to reach the peak of the trace is not altered in the cecum of Nlgn3 mice compared to WT mice. (**G**) The increase in current, ΔI_SC_ is significantly lower in the cecum of Nlgn3 mice compared to WT mice. (**H**) Representative current traces versus time of increase in I_SC_ in WT (blue) and Nlgn3 (orange). Statistical comparisons were conducted using Student’s unpaired *t-*test and individual data and mean ± SD were plotted for n = 11 mice in each group. ns = p > 0.05, *p < 0.05. WT = wildtype; Nlgn3 = *Nlgn3*^*R451C*^.

Given our previous finding of an increase in the number of NOS-labelled neurons and total neurons in the cecal submucosal plexus in *Nlgn3*^*R451C*^ mice^38^, we studied gut secretion and permeability in the cecum. We found no differences in cecal transepithelial resistance (TER) between WT and *Nlgn3*^*R451C*^ mice (Fig. 5A). Similarly, there was no difference in apparent permeability in the cecum of WT and *Nlgn3*^*R451C*^ mice (Fig. 5B). Cecal TER in wildtype and *Nlgn3*^*R451C*^ mice was comparable over time and in response to FITC-Dextran (Fig. 5C). The concentration of FITC traversing cecal tissue preparations over time was also comparable between WT and *Nlgn3*^*R451C*^ mice (Fig. 5D).

Next, we investigated changes in short-circuit current (I_SC_) in response to application of the nicotinic receptor antagonist DMPP as an index of electrogenic secretion. At baseline, I_SC_ was not significantly different between WT and *Nlgn3*^*R451C*^ cecum samples (Supp. Fig. 1A). Following application of DMPP (10 µM), both WT and *Nlgn3*^*R451C*^ cecum samples showed a similar magnitude of increased short-circuit current (ΔI_SC_) (Supp. Fig. 1B,C). The time to reach the peak DMPP response was similar for WT and *Nlgn3*^*R451C*^ preparations (Supp. Fig. 1D). To substantiate these findings further, these experiments were repeated with a higher concentration of DMPP (20 µM). Although both the reduction of I_SC_ at baseline (Fig. 5E) and response time in the presence of 20 µM DMPP were similar for WT and *Nlgn3*^*R451C*^ samples (Fig. 5F), a significant decrease in the maximal I_SC_ response was observed in *Nlgn3*^*R451C*^ compared to WT samples (Fig. 5G,H) (data presented in Supp. Table 4).

### VIP and ChAT submucosal neuronal numbers are unchanged in the cecum of *Nlgn3*^*R451C*^ mice

**Figure 6:**
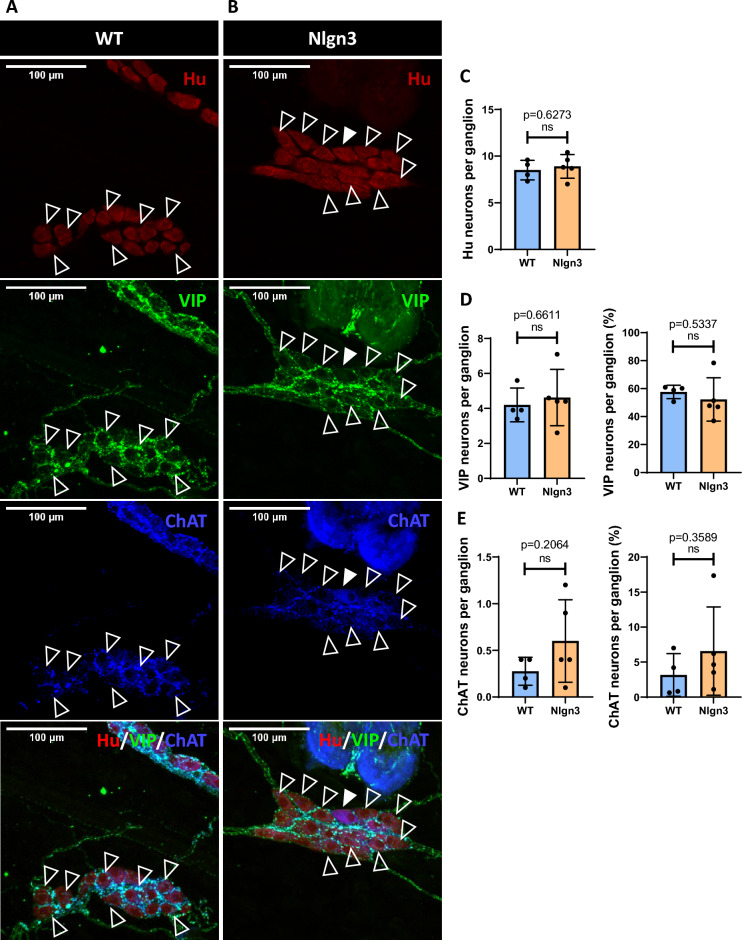
Hu, VIP, and ChAT immunostaining in the submucosal plexus of WT (**A**) and *Nlgn3*^*R451C*^ (**B**) mice. VIP (clear arrowheads) and ChAT (filled arrowhead) immunolabelled enteric neurons in the cecal submucosal plexus are pictured. (**C**) No significant difference was observed in the number of Hu immunolabelled neurons per ganglion. (**D**) The number of VIP immunostained neurons and the percentage of VIP immunostained neurons per ganglion was not significantly different. (**E**) No significant difference was observed in the number of ChAT immunolabelled neurons and the percentage of ChAT neurons per ganglion. Statistical comparisons were conducted using Student’s unpaired *t-*test and individual data and mean ± SD were plotted for n = 11 mice in each group. ns = p > 0.05. WT = wildtype; Nlgn3 = *Nlgn3*^*R451C*^.

Vasoactive intestinal peptide (VIP) and acetylcholine (ACh) are key neurotransmitters of secretomotor neurons essential to the neural control of secretion^42^. The choline acetyltransferase (ChAT) enzyme is directly involved in acetylcholine synthesis and its expression delineates cholinergic neurons. Since dose-dependent changes in neurally-evoked secretion were observed in *Nlgn3*^*R451C*^ cecum following DMPP exposure (Fig. 5G,H), we analysed the number of submucosal neurons immunolabelled for VIP and ChAT (Fig. 6A,B). In contrast with our previous findings^38^, we did not detect a significant change in the total numbers of neurons per ganglion in the submucosal plexus of *Nlgn3*^*R451C*^ cecum compared to WT mice (Fig. 6C). This discrepancy may be due to variations in animal supply between the animal facilities used for the two studies. Specifically, our previous work utilized mice bred at RMIT University Animal Facility, while the current study employed mice bred at The University of Melbourne Biomedical Sciences Animal Facility. These differences in breeding and housing conditions could potentially influence the observed outcomes. Our analysis of neuronal subtypes showed similar numbers and proportions of VIP (Fig. 6D) and ChAT (Fig. 6E) immunolabelled neurons per ganglion in the submucosal plexus of WT and *Nlgn3*^*R451C*^ cecal samples (Supp. Table 5).

### Expansion of gut-associated lymphoid tissue (cecal patches) in *Nlgn3*^*R451C*^ cecum

**Figure 7:**
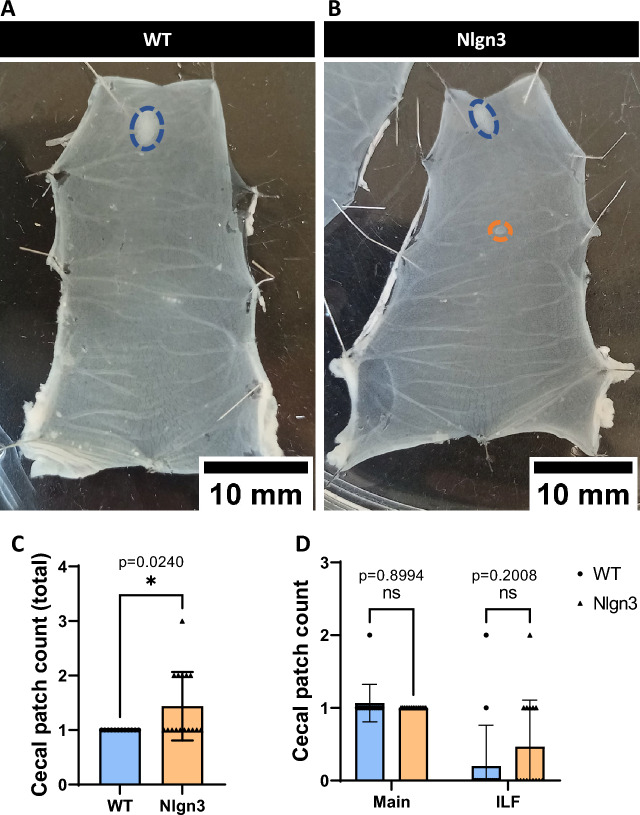
Multiple cecal patches are present in *Nlgn3*^*R451C*^ mice.Representative images of pinned-out cecum from WT (**A**) and Nlgn3 (**B**) mice. Blue dashes outline the main cecal patch, whereas orange dashes outline the isolated lymphoid follicles (ILF) typically found along the body of the cecum. (**C**) Total cecal patch numbers are significantly higher in Nlgn3 cecum compared with WT littermates. 3 outliers were removed in the WT group according to the ROUT Q = 1% method. (**D**) When total cecal patch count is split into main and ILF cecal patches, the difference is not significant despite an evident, qualitative trend for an increase in Nlgn3 ILF. Statistical comparisons were conducted using Student’s unpaired *t-*test and individual data and mean ± SD were plotted for WT: n = 12; Nlgn3: n = 16 mice in each group. ns = p > 0.05, *p < 0.05. WT = wildtype; Nlgn3 = *Nlgn3*^*R451C*^.

The cecal patch is an aggregate of gut-associated lymphoid tissue (GALT) located at the blind end of the cecum. We previously reported dysmorphic gut macrophages within the *Nlgn3*^*R451C*^ cecal patch, indicative of a reactive immune phenotype^38^. Here, we studied the gross morphology of the cecal patch. Typically, a single primary cecal patch is located at the tip of the cecal pouch (Fig. 7A), however, additional smaller patches (also referred to as isolated lymphoid follicles; ILF) were more commonly observed in the *Nlgn3*^*R451C*^ cecum (Fig. 7B,D). These patches are smaller in size than a primary cecal patch and possess a single germinal centre with an overlying layer of follicle-associated epithelium. In the cecum, ILFs are typically located opposite to the mesenteric border in-line with the main patch along the cecal body. Interestingly, *Nlgn3*^*R451C*^ cecal samples had a greater number of total cecal patches compared to WT (Fig. 7C).

**Figure 8:**
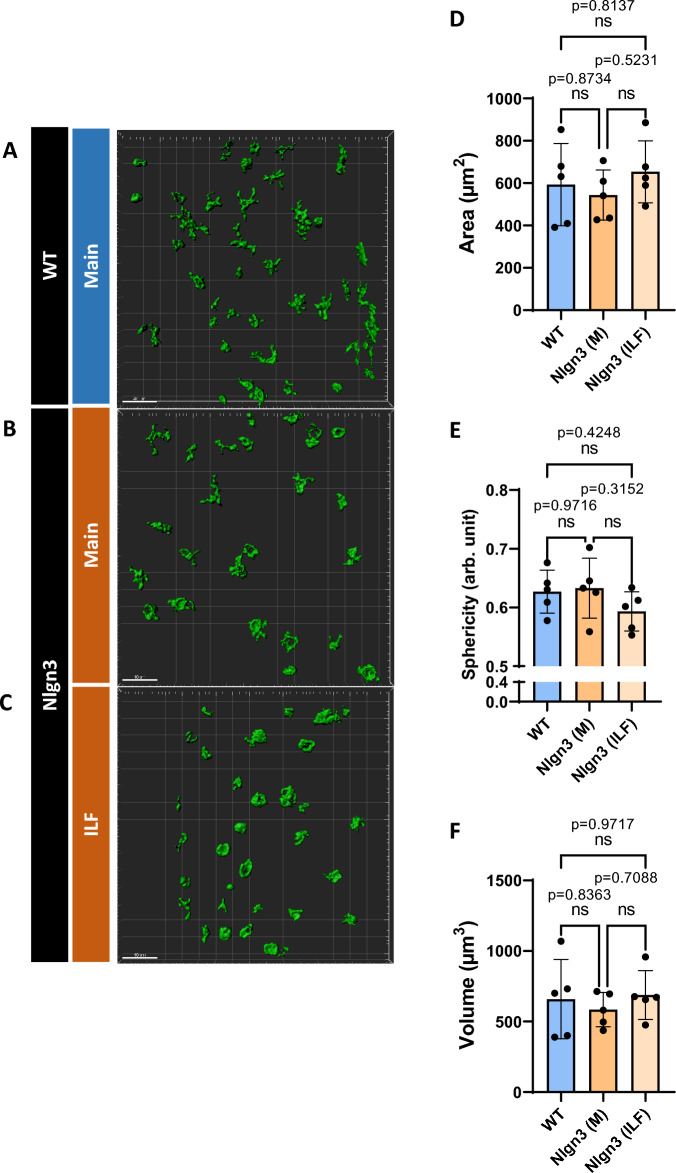
No morphological differences documented in CD11c^+^ cells within cecal patches of *Nlgn3*^*R451C*^ and WT mice. Both main and ILF *Nlgn3*^*R451C*^ cecal patches (**A,B**) were immunostained for CD11c whereas only main patches were immunostained for WT mice (**C**). No significant morphological differences were observed among WT main, Nlgn3 main, and Nlgn3 ILF cecal patches with the parametrized area (**D**), sphericity (**E**), and volume (**F**). Statistical comparisons were conducted using one-way ANOVA with Tukey’s multiple comparisons test. Individual data and mean ± SD were plotted for n = 5 mice in each group. ns = p > 0.005. WT = wildtype; Nlgn3 = *Nlgn3*^*R451C*^.

Tissue from main cecal patches were processed for WT mice (Fig. 8A), whereas both main and ILF cecal patches for the *Nlgn3*^*R451C*^ group were processed for cellular analyses (Fig. 8B,C). Following 3D reconstruction and analysis of CD11c^+^ dendritic cells of WT and *Nlgn3*^*R451C*^ cecal patch tissue, we found no morphological differences in cellular area (Fig. 8D), sphericity (Fig. 8E), or volume (Fig. 8F) suggesting that although there is an expansion of GALT in *Nlgn3*^*R451C*^ mice, the overall tissue structure is normal.

**Figure 9:**
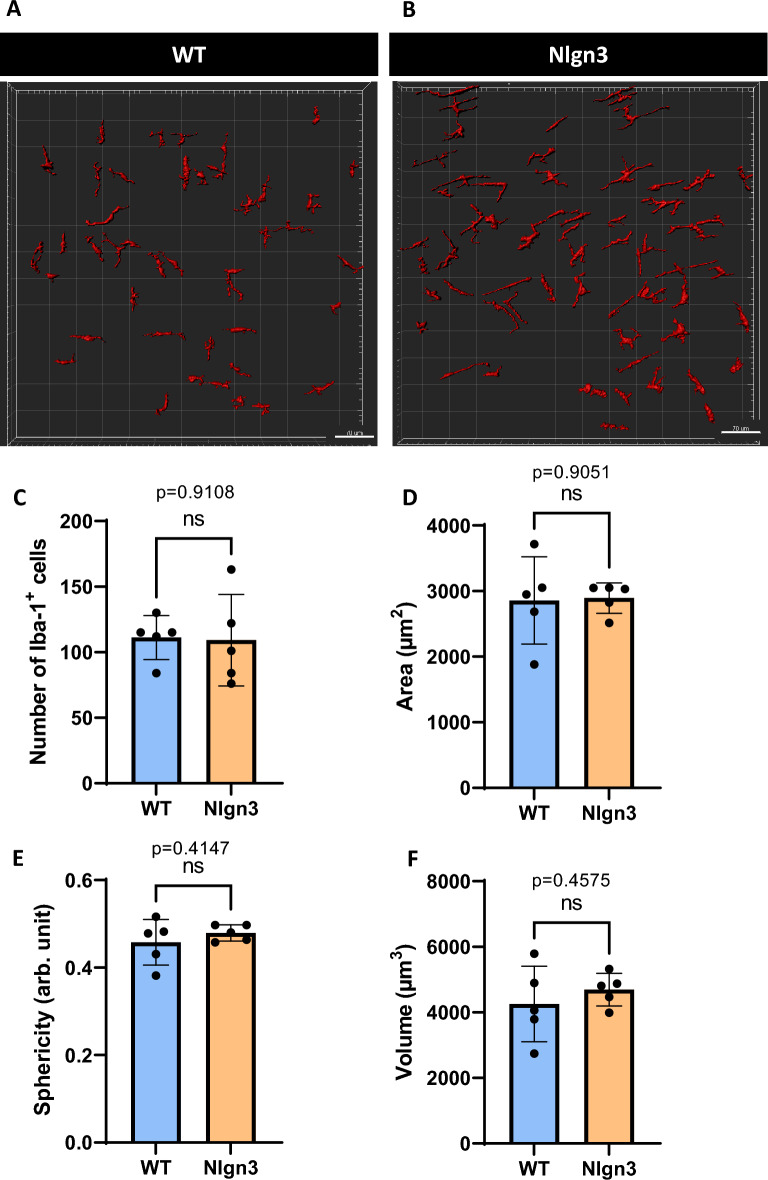
No morphological difference was observed in Iba-1^+^ immunopositive cells in the myenteric plexus of *Nlgn3*^*R451C*^ cecum compared to WT littermates. 3D reconstruction and analysis of Iba-1^+^ cells in WT (**A**) and Nlgn3 (**B**) myenteric plexus. No significant morphological differences were observed with the parametrized batches of Iba-1^+^ cells for (**C**), area (**D**), sphericity (**E**), and volume (**F**). Statistical comparisons were conducted using Student’s unpaired *t-*test. Individual data and mean ± SD were plotted for n = 5 mice in each group. ns = p > 0.05. WT = wildtype; Nlgn3 = *Nlgn3*^*R451C*^.

Immunolabelled Iba-1^+^ macrophages within the myenteric plexus were analysed for morphological differences as an indicator of immune reactivity (Fig. 9A,B). In contrast with findings in the cecal patch^38^, we observed similar numbers of Iba-1^+^ cells in the cecal myenteric plexus of *Nlgn3*^*R451C*^ compared to WT littermates (Fig. 9C). A 3D analysis similarly showed no significant morphological differences in Iba-1^+^ cellular area (Fig. 9D), sphericity (Fig. 9E) or volume (Fig. 9F) (data summarized in Supp. Table 6). Similar to our findings when analysing CD11c^+^ dendritic cells, we showed that although there is an increased number of cecal GALT aggregates, the density and morphology of Iba-1^+^ cells remain unchanged in *Nlgn3*^*R451C*^ mice.

## Discussion

Neurodevelopmental disorders are associated with GI symptoms^11,16,43,44^, with dysmotility likely being a common factor. Such phenotypic features are recapitulated in preclinical models, notably in mice models of disorders such as autism^34,45^. Abnormalities in cecal weight and microbial populations are typically reported in a myriad of animal models including germ-free^46,47^, antibiotic-treated^48^ and models of inflammation and neurological diseases^38,49^, yet in the *Nlgn3*^*R451C*^ mouse cecum, a detailed characterisation of cecal motility in combination with GALT structural analysis has not been reported.

First, we investigated mouse cecum motility and document CeMCs, reminiscent of colonic migrating motor complexes (CMMCs), that largely comprised multidirectional contractions, a feature that is similar to contractions documented in rabbit cecum^50^. We report that most cecal contractile activity was abolished in the presence of TTX, indicating that these contraction patterns in the cecum are neurogenically driven. Like the colon, the contraction pattern in the body of the cecum was largely abolished when TTX was added to the organ bath. Although some residual activity appears to persist, this corresponds to the ileocecal junction in the cecal preparation which may serve as a transitional zone between ileum and cecal motility patterns. This could indicate that a small but significant subpopulation of enteric neurons in that area are insensitive or resistant to TTX. The presence of TTX-resistant Na_v_1.9 channels has previously been reported in myenteric neurons in mice, rats, and guinea pigs^51–54^, and may play a role in CeMCs, as observed in previous studies on CMMCs^55^. Interestingly, Copel and colleagues demonstrated that CMMCs do occur in Na_v_1.9 null mice, but their characteristics (frequency, duration, and propagation direction) differ significantly from controls^55^. We also compared our cecal motility heatmaps and graphs obtained with data obtained from the mouse jejunum by Neal et al.^56^, and did not observe a corresponding high frequency peak at 0.70–0.75 Hz at control or in the presence of TTX in the cecum. Thus, we conclude that cecum motility in mice is more similar to the colon than the jejunum. This finding is consistent with the cecum's classification as part of the large intestine in mammals, despite its anatomical differences.

Our study demonstrated that CeMCs can serve as a useful phenotypic marker for both normal GI functions and abnormal states. Our findings showed that mice harbouring the autism-associated *Nlgn3*^*R451C*^ mutation had increased CeMC frequencies and shorter CeMC durations. It is plausible that cecal dysmotility decreases the efficiency of contractile activity in *Nlgn3*^*R451C*^ mice, leading to reduced luminal content volumes in the cecum which, in turn, underly a decrease in cecal weight.

We found that cecal dysmotility in *Nlgn3*^*R451C*^ mice led to changes in the frequency of downstream contractions in the proximal colon. Due to size limitations of the organ bath, the ileo-cecal-colonic preparation dissected for cecal motility experiments comprised only the proximal colon region. It remains to be determined as to whether these proximal colonic contractions can migrate more than half the length of the colon and could therefore be considered a colonic migrating motor complex (CMMC)^57^. Nevertheless, we found cecal-colonic contraction intervals to be shorter in *Nlgn3*^*R451C*^ mice compared to wildtype, suggesting that colonic function is affected in these mutant mice, likely leading to defective water absorption and fecal pellet formation. Previous studies have demonstrated that *Nlgn3*^*R451C*^ mice exhibit a reduction of approximately 10% in Nlgn3 protein levels at post-synaptic membranes, resulting in increased inhibitory neuronal transmission in the central nervous system (CNS)^24,28^. Within the gastrointestinal tract, Nlgn3 mRNA is expressed in myenteric and submucosal neurons^58^. Given the crucial role of intrinsic neural circuits within the ENS, which comprises sensory neurons, excitatory and inhibitory motor neurons, and ascending and descending interneurons responsible for modulating intestinal motility and motor behaviours^59^, the presence of the *Nlgn3*^*R451C*^ mutation may disrupt the activity of this motor neural circuit, potentially explaining the observed caecal dysmotility. Nlgn3 mRNA expression is also detected in enteric glia, with a specific decrease observed in myenteric glia due to the *Nlgn3*^*R451C*^ mutation^58^. Enteric glial cells have been highlighted for their involvement in regulating intestinal motility^60,61^, and could also contribute to alter caecal motility described in this work. Enteric glial cells also directly respond to immunomodulatory signals which enables neuro-immune crosstalk with enteric neurons and immune cells in the gastrointestinal tract^62^.

In contrast with clinical reports of individuals with autism presenting with increased gut permeability or impaired intestinal barrier integrity^63–65^, we did not observe significant differences in cecal transepithelial resistance (TER) or permeability in *Nlgn3*^*R451C*^ mice. Basal short-circuit current was unchanged in Nlgn3^R451C^ mice, indicating that, at resting physiological conditions, mucosal secretion is not affected at neuronal synapses. When submucosal neurons were selectively stimulated with DMPP, however, *Nlgn3*^*R451C*^ cecal tissue showed a significantly reduced peak secretory response compared to WT. This finding suggests that the *Nlgn3*^*R451C*^ mutation reduces nicotinic acetylcholine receptor (nAChR) activity given that DMPP acts exclusively on this receptor subtype. Therefore, in future studies, it may be valuable to investigate the expression of AChRs in the cecum using methods such as qPCR or Western blotting. The intestinal mucosal secretory system is vital for secreting water and mucus and flushing foreign pathogens out of the gastrointestinal lumen. Decreased secretory function could thus contribute to the observed decrease in cecal weight due to reduced water content and may also cause defective pathogen removal. We found no significant difference in the proportions of ChAT and VIP-stained secretomotor neurons. In summary, our findings suggest that the autism-associated Neuroligin-3 R451C mutation alters gastrointestinal secretory function via a nAChR-mediated pathway in the submucosal plexus.

The mouse cecum typically possesses one prominent lymphoid tissue aggregate at the apex of the blind-ended pouch^66^. Here, we identified significantly more cecal patches in *Nlgn3*^*R451C*^ mice compared to WT littermates. Although IgA-secreting cells are present in both cecal and Peyer’s patches, IgA^+^ cells from the cecal patch that migrate to the colon can alter microbiota composition, a phenomenon that has not been described for Peyer’s patches^7^. Based on our findings, the composition of cecal and colonic microbial communities and their function may be altered in *Nlgn3*^*R451C*^ mice and warrants further investigation.

Given the increased number of cecal patches in *Nlgn3*^*R451C*^ mice, we investigated cecal patch immune profiles in these mice. CD11c is present on many immune cells including macrophages but has historically been considered as a marker for dendritic cells. Antigen-presenting dendritic cells localized in the GALT are important for producing IgA in response to commensal bacteria within the lumen^67,68^. Although no difference was observed in CD11c-immunolabeled dendritic cells in cecal patches or myenteric Iba-1 macrophages, other immune cell types may still be affected. Therefore, to determine which immune cell populations are involved, it may be necessary to examine a broad range of immune cell profiles using techniques such as flow cytometry.

Here, we show that an ASD-associated missense mutation in the gene encoding Neuroligin-3 causes gastrointestinal dysfunction by impacting cecal motility, secretory function, and gut-associated lymphoid tissue structure in mice. To date, the Neuroligin-3 protein has been identified in mouse colon tissue^69^ and our recent findings indicate that Neuroligin-3 mRNA is present in enteric neurons and glia in mice^58^. The contribution of Neuroligin-3 to the development and function of the cecum is currently unknown. The potential existence of multiple ASD-relevant mutations that also induce gastrointestinal dysfunction may indicate a broader biological mechanism underlying gut symptoms in individuals with ASD. As such, targeting cecal dysfunction could support therapeutic strategies to alter metabolite profiles and improve gastrointestinal symptoms in ASD. Further research is needed to elucidate the precise role of Nlgn3 and other ASD-associated mutations in cecal function and to explore the therapeutic potential of targeting this pathway.

## Methods

### Animals

Adult male *Nlgn3*^*R451C*^ mice and wildtype littermates (8–14 weeks old) were bred and maintained on a C57BL/6J background at The University of Melbourne Biomedical Sciences Animal Facility and Royal Melbourne Institute of Technology (RMIT) University Animal Facility. The use of male mice is justified as follows: (1) ASD predominantly affects males; (2) The *Nlgn3*^*R451C*^ mutation is located on the X-chromosome. Using the current breeding strategy, only male mice demonstrate the full effect of the missense mutation. Future projects will aim to assess any potential effects of the *Nlgn3*^*R451C*^ mutation in females. Experimental mice were transported to RMIT Animal Facility and culled by cervical dislocation at 8 weeks of age under approved ethical guidelines. All experiments were performed in accordance with strict guidelines and regulations as approved by the Royal Melbourne Institute of Technology (RMIT) University Animal Ethics Committee (Animal Ethics Number: 1727). All experiments complied with the Animal Research: Reporting of In Vivo Experiments (ARRIVE) guidelines 2.0.

### Ex vivo cecal motility assay

Cecal motility was studied ex vivo to visualise mouse cecum motility patterns. This approach is derived from a colon motility assay^70^ combined with a tri-cannulation technique previously reported in rabbit cecum^39,50^, as follows. Freshly dissected caeca were cannulated in an organ bath of Krebs physiological saline (118 mM NaCl, 4.6 mM KCl, 2.5 mM CaCl_2_, 1.2 mM MgSO_4_, 1 mM NaH_2_PO_4_, 25 mM NaHCO_3_, 11 mM D-glucose; aerated with 95% O_2_, 5% CO_2_; superfused at a flow rate of 8 ml min^−1^; at 36 °C). The ileal end was connected to a reservoir filled with saline, and the cecal tip and colon end connected to outflow tubes. Front and back pressure of approximately 4 cm of H_2_O was established^70^. Video recordings were filmed via Virtual Dub video capture software (version 1.10.4, open source) with a Logitech webcam (QuickCam Pro 4000; I-Tech, Ultimo, NSW, Australia) mounted at a fixed height directly above the organ bath. Video files (15-min duration) were then converted to spatiotemporal heatmaps using purpose-built, in-house open-source software (Analyze2; Swaminathan et al.^70^), with pixel hue representing the diameter of the cecal preparation; cooler colours (blue, green) denoting wider diameter (relaxed) gut, while warmer colours (red, yellow) denote narrow diameter (constricted) gut. The *x*-axes of heatmaps represent time while the *y*-axis plots the position along the length of the cecal segment (Fig. 1A). A protocol comprising a 30-min equilibration period followed by four 15-min (totalling 1 h duration) recordings was conducted to record spontaneous cecal contractions. To further characterize cecal contractile patterns, tetrodotoxin (TTX; Sigma-Aldrich, St Louis, MO, USA), a Na^+^ channel blocker, was applied to the organ bath to a final concentration of 1 μm.

Spatiotemporal heatmaps were used to assess potential differences in neurally-mediated cecal motor activities, namely Cecal Motor Complexes (CeMC) and gut resting diameter. Similar nomenclature is used for colonic migrating motor complexes (CMMCs), which are regular neurally-regulated contractions that propagate along the colon^41^. Data obtained from each spatiotemporal heatmap include: CeMC frequency, velocity, duration, and period of quiescence between each CeMC. We additionally identified the proportion of CeMCs with a canonical forward-reverse contraction pattern, as well as cecal-colonic contraction intervals (defined as the time elapsed between the start of a CeMC and corresponding contraction in the attached colon tissue segment visible on heatmaps). Resting gut diameter was measured during the quiescence interval between CeMCs while in constant intraluminal pressure by measuring cross sections of the heatmap corresponding to various cecal regions: the cecal ampulla (measured at a point corresponding to the first 20% of cecal length), mid-cecum (60%), and cecal tip (80%).

### Cecal content, histology, and mucus content analysis

Cecal samples were weighed upon dissection then cut open and contents flushed out with saline. The cecal tissue was then re-weighed, with the difference in readings representing cecal content weight, further standardized between mice against body weight.

For mucus staining, cecum samples were first fixed in methanol-Carnoy’s fixative (composition, V/V: 60% absolute methanol, 30% chloroform, 10% glacial acetic acid) overnight at 4 °C. Samples were washed twice in absolute methanol (30 min each), twice in absolute ethanol (20 min each) and processed in two rounds of xylene (15 min each). Tissue was then paraffin-embedded and sectioned at 4 µm thickness. Sections were stained with 1% Alcian blue in 3% Acetic Acid (pH 2.5) (Sigma-Aldrich, St Louis, MO, USA) for 10 min and counterstained with Nuclear Fast Red (Sigma-Aldrich, St Louis, MO, USA) for 5 min. Tissue processing and staining steps were performed by the Biomedical Sciences Histology Facility at The University of Melbourne (Parkville, Australia). Sections were imaged on a Slide Scanner Microscope (Olympus Australia Pty. Ltd.; Melbourne, Australia), with mucus content in five cross sections per cecum quantified using FIJI ImageJ (ImageJ 1.52a, NIH)^71^. Areas of Alcian Blue mucus stained were selected using the colour threshold feature of FIJI Image J and normalized against the total area of the cecum cross section.

### Permeability and secretion assays

A 4-chamber P2400 EasyMount Ussing Chamber system (Physiologic Instruments, San Diego, CA, USA) was connected to electrodes using salt agar bridges (electrode tips filled with 2.5% agarose/3 M KCl solution) and to an EC-825A Epithelial Voltage Clamp amplifier (Warner Instruments, Holliston, MA, USA). The Ussing chamber was initially calibrated by compensating for fluid resistance and checking for asymmetries in the recording electrodes according to the manufacturer’s instructions. Under stereomicroscopy (Olympus, SZ61, Japan), the serosa, longitudinal muscle and circular muscle layers of the cecum were carefully peeled away to obtain mucosa-submucosa preparations, each mounted on Ussing chamber slider. Each side of the chamber was superfused with 5 mL of Krebs solution and carbogenated throughout the experiment. Cecum preparations were equilibrated for 20 min prior to collecting measurements, as follows. Transepithelial resistance (TER) was measured by applying a voltage-clamp of 1 mV and recording the resulting short-circuit current (I_SC_) reading. This procedure was repeated 5 times at 50 s intervals to obtain an average reading. The TER was finally calculated using Ohm’s Law (Voltage = Current x Resistance; V = IR). Paracellular permeability of gut tissue samples was assessed by applying fluorescein isothiocyanate-dextran (10 mg/mL 4 kDa FITC-dextran, Sigma-Aldrich, St Louis, MO, USA) to the mucosal compartment, to a final concentration of 0.5 mg/mL. At each timepoint, 200 µL of solution was collected from the serosal compartment and absorbance readings documented on the FlexStation 3 Microplate Reader (Molecular Devices, Sunnyvale, CA, USA; Excitation 485 nm; Emission 538 nm), with concentrations calculated based on a standard curve. TER readings were taken at 15-min intervals to assess for changes upon addition of FITC-dextran into the system.

Electrogenic secretion of the cecal preparation was assessed by measuring short-circuit current (I_SC_) in the presence of a ganglion-specific nicotinic acetylcholine receptor (nAChR) agonist, 1,1-dimethyl-4-phenylpiperazinium (DMPP)^72^. Transepithelial voltage was initially clamped at 0 mV. The subsequent short-circuit current (I_SC_) was monitored at baseline and compared with when DMPP was added to the serosal compartment of the chamber (where submucosal neurons are exposed). A non-specific cholinergic agonist, Carbachol, was added to the chamber’s serosal compartment at the conclusion of each experiment to evoke a significant increase in I_SC_ as an indicator of tissue viability. Experiments with tissues nonresponsive to Carbachol at this step were discarded. The duration of each recording experiment was restricted to 3 h.

### Microdissection and immunofluorescence staining

Cecal tissues were pinned flat and fixed in 4% formaldehyde (Sigma-Aldrich, Germany) overnight at 4 °C. The cecal patch was carefully excised and stored in 30% sucrose overnight at 4 °C for cryoprotection before embedding in Tissue-Tek O.C.T. compound (Sakura Torrance, CA, USA) for cryosectioning at 10 µm thickness. Myenteric plexus wholemount preparations were obtained by peeling the mucosal and circular muscle layers of pinned-out cecum fixed in 4% formaldehyde. Cecal submucosal plexus preparations were obtained by carefully separating the mucosal layers from underlying muscular layers and gently scraping off the upper villi layer of cecal tissue fixed in 4% paraformaldehyde. Both wholemount preparations and sections were incubated with pre-immune serum and stained as per described in Supp. Table 1.

### Analysis of neurons and immune cell populations

Cecal neurons from the myenteric and submucosal plexus were analysed using Image J software (ImageJ 1.52a, NIH, Bethesda, MD, USA). Ten ganglia from each preparation were randomly selected and neurons counted for their respective immune-labelled signals. Imaris software (Imaris 64X 9.1.0; Bitplane AG, UK) was used for 3D image reconstruction and analysis of immunolabelled Iba-1^+^ and CD11c^+^ cells.

### Statistical analysis

GraphPad Prism version 9.1.2 for Windows (GraphPad Software, San Diego, USA) was used to perform statistical analyses. CeMC frequency from motility assays was analysed using a Mann–Whitney U statistical test. All measures of cecal motility were assessed using the Benjamini–Hochberg procedure to account for false discovery rate. Remaining data were analysed using Student’s unpaired *t*-tests, one-way ANOVA with Tukey’s multiple comparisons test, and two-way ANOVA statistical tests with Šídák multiple comparisons test where appropriate. Statistical significance was taken as p < 0.05. Data are presented as mean ± standard deviation (SD).

### Preprint server

A version of this manuscript has been uploaded to the BioRxiv preprint server^73^.

### Supplementary Information

Supplementary Video 1.

Supplementary Information 1.

## Data Availability

The datasets generated and analysed during the current study are available in the RMIT University Figshare repository (https://doi.org/10.25439/rmt.21235755).
