# Supplementary material for: Impaired cecal motility and secretion alongside expansion of gut-associated lymphoid tissue in the Nlgn3R451C mouse model of autism
Source: Sci Rep. 2023 Aug 4;13:12687. doi: 10.1038/s41598-023-39555-y (PMC10403596; doi:10.1038/s41598-023-39555-y)
Supplement: Supplementary file 2 — Supplementary Information 1. [file 41598_2023_39555_MOESM2_ESM.pdf]

Impaired cecal motility and secretion alongside expansion of gut-associated lymphoid tissue in the Nlgn3^{R451C} mouse model of autism

Short title: Cecal dysfunction in Nlgn3^{R451C} mice

Chalystha Yie Qin Lee[1], Gayathri K. Balasuriya[2], Madushani Herath[3, 4, 5], Ashley E. Franks[6], Elisa L. Hill-Yardin[1, 5*]

[1] School of Health and Biomedical Sciences, RMIT University, Bundoora, VIC, Australia;
[2] Graduate School of Medicine, Kobe University, Kobe, Japan;
[3] Department of Pathology and Immunology, Baylor College of Medicine, Houston, TX, United States;
[4] Texas Children's Microbiome Center, Department of Pathology, Texas Children's Hospital, Houston, TX, United States;
[5] Department of Physiology, University of Melbourne, Parkville, VIC, Australia;
[6] School of Life Sciences, La Trobe University, Bundoora, VIC, Australia.
[*]Corresponding author.

Correspondence
Elisa Hill-Yardin, School of Health and Biomedical Sciences, RMIT University, Building 223, Bundoora West Campus, 225-245 Clements Drive, Bundoora VIC 3083 Australia, elisa.hill@rmit.edu.au, +61 4 3405 2127

Supplementary Figure 1

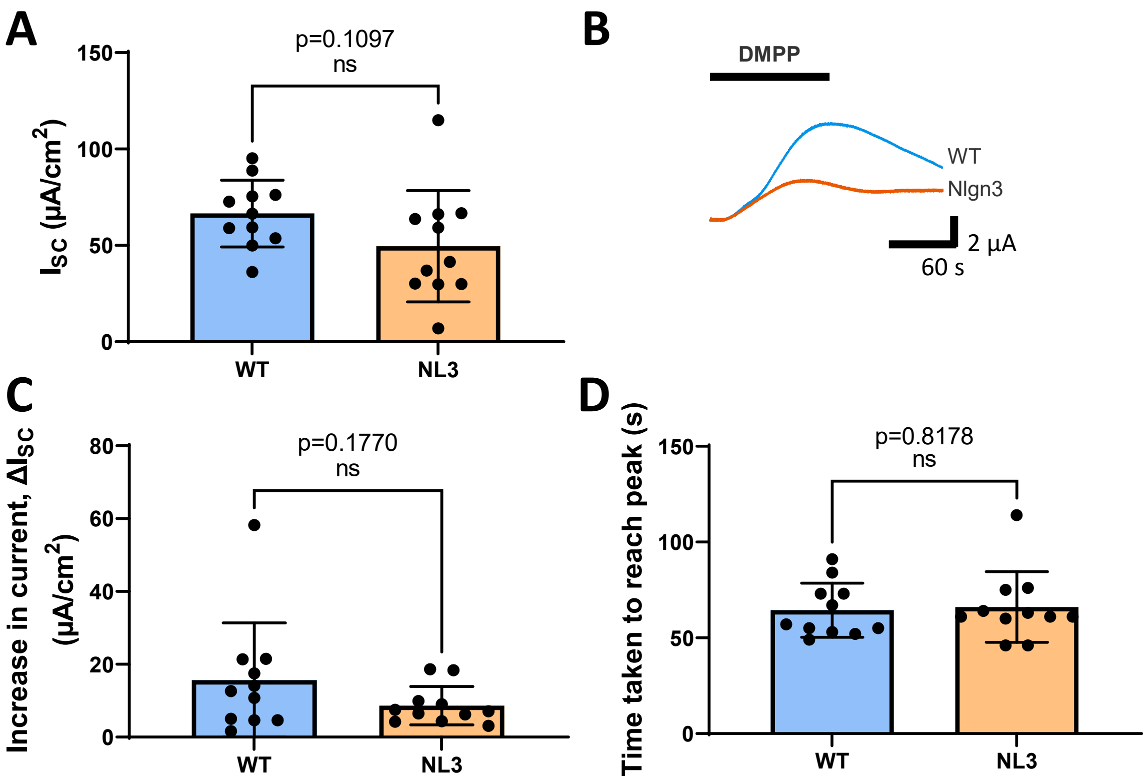

Supplementary Figure 1: Short-circuit current, I_{SC}, response to 10 µM DMPP. A: The I_{SC} at baseline is not significantly different in $Nlgn3^{R451C}$ compared to WT. B: Representative current traces versus time of increase in I_{SC} in WT (blue) and Nlgn3 (orange). C: The increase in current, ΔI_{SC} and D: time taken to reach the peak of the trace is not altered in the cecum of Nlgn3 mice compared to WT mice. Statistical comparisons were conducted using Student's unpaired *t*-test. Individual data and mean ± SD were plotted for n = 11 mice in each group. ns = $p > 0.05$.

Supplementary video 1

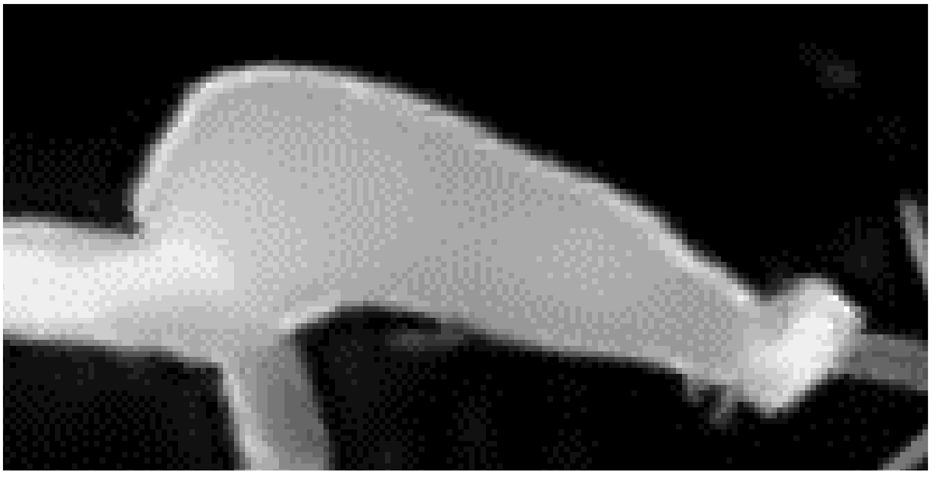

Supplementary Video 1: A real-time video recording of contractions in the cecum.

Supplementary Table 1: Concentrations of primary and secondary antibody steps.

		Caecal patch cross-section	Myenteric plexus			Submucosal plexus		
Primary antibody	**Antibody**	CD11c-647	Hu	NOS	Iba1	Hu	VIP	ChAT
	**Host**	Armenian Hamster	Human	Sheep	Rabbit	Human	Rabbit	Goat
	**Dilution**	1:500	1:5000	1:1000	1:400	1:5000	1:100	1:100
	**Source**	Biolegend	Mayo Clinic *	Sigma-Aldrich	Fujifilm Wako	Mayo Clinic *	Immunostar	Sigma-Aldrich
	**Cat. No.**	117314	-	AB1529	019-19741	-	20077	ab144P
Secondary antibody	**Target**	-	Human	Sheep	Rabbit	Human	Rabbit	Sheep
	**Fluorophore**		594	488	647	594	488	647
	**Host**		Donkey	Donkey	Donkey	Donkey	Donkey	Donkey
	**Dilution**		1:750	1:400	1:400	1:750	1:400	1:400
	**Source**		Jackson Immuno Research	Invitrogen	Jackson Immuno Research	Jackson Immuno Research	Invitrogen	Invitrogen
	**Cat. No.**		709-585-149	A11015	711-605-152	709-585-149	2289872	A21448

*Hu antibodies were a gift from Dr. Vanda Lennon, Mayo Clinic, Rochester, MN.

Supplementary Table 2: Analysis of CaeMCs including forward/pro-peristaltic and reverse/anti-peristaltic contraction parameters.

Overall CaeMC parameters					
Parameters	WT (mean ± SD)	NL3 (mean ± SD)	p-value	Benjamini-Hochberg Adjusted p-value	Significance
No. of CaeMC in 15min	17.45 ± 3.42	24.09 ± 6.41	0.0103	0.0283	*
CaeMC velocity (mm/s)	1.475 ± 0.578	2.200 ± 0.884	0.037	0.0678	NS
CaeMC duration (s)	20.77 ± 8.10	11.59 ± 5.15	0.0048	0.0211	*
CaeMC quiescence period (s)	25.21 ± 6.83	26.21 ± 6.95	0.7363	0.8099	NS
Percentage of F-R CaeMC (%)	78.11 ± 9.06	55.73 ± 13.2	0.0001	0.0022	**
Caecal-colonic contraction interval (s)	19.61 ± 7.47	8.476 ± 3.54	0.0002	0.0022	**
Forward / Pro-peristaltic Contractions (FC)					
FC velocity (mm/s)	4.125 ± 1.01	6.995 ± 3.64	0.0202	0.0494	*
FC duration (s)	3.795 ± 1.06	2.119 ± 0.852	0.0006	0.0044	**
FC Start position (%)	92.17 ± 5.63	81.17 ± 8.81	0.0023	0.0127	**
Reverse / Anti-peristaltic Contractions (RC)					
RC velocity (mm/s)	7.94 ± 1.72	9.82 ± 4.21	0.1857	0.2724	NS
RC duration (s)	1.89 ± 0.386	1.37 ± 0.443	0.0082	0.0301	*

Supplementary Table 3: Analysis of caecal content and mucus content.

Caecal content				
Parameters	WT (mean ± SD)	NL3 (mean ± SD)	p-value	Significance
Caecal weight (before clearing) (g)	0.5564 ± 0.0776	0.4717 ± 0.0766	0.0101	*
Weight of content (g)	0.3921 ± 0.0728	0.3175 ± 0.0605	0.0096	**
Weight of content/Body weight	0.01410 ± 0.00265	0.01214 ± 0.00204	0.0484	*
Mucus content				
Mucus Area ($\mu m^2/\mu m^2$)	0.1023 ± 0.0605	0.07409 ± 0.0462	0.2564	NS
Overall Caecal Area ($x10^8$ μm^2)	1.208 ± 0.403	1.127 ± 0.159	0.5592	NS

Supplementary Table 4: Analysis of Ussing chamber parameters and response to DMPP.

Overall Ussing chamber parameters				
Parameters	WT (mean ± SD)	NL3 (mean ± SD)	p-value	Significance
Transepithelial resistance (TER) ($\times 10^3$ Ω/cm^2)	0.2306 ± 0.0729	0.2621 ± 0.102	0.1809	NS
TER after FITC ($\times 10^3$ Ω/cm^2)	0.4056 ± 0.0835	0.4828 ± 0.119	0.7630	NS
Concentration of FITC (ng/ml)	21.14 ± 25.6	23.94 ± 29.8	0.2518	NS
Apparent permeability, P_{app} (ng/ml/min/cm^2)	2.758 ± 1.05	3.204 ± 0.498	0.2400	NS
Response to 10 µM DMPP				
Short-circuit current, I_{SC} (µA/cm^2)	66.56 ± 17.3	49.57 ± 28.9	0.1097	NS
Increase in current, ΔI_{SC} (µA/cm^2)	15.64 ± 15.7	8.636 ± 5.27	0.1770	NS
Time taken to reach peak (s)	64.45 ± 14.1	66.09 ± 18.5	0.8178	NS
Response to 20 µM DMPP				
Short-circuit current, I_{SC} (µA/cm^2)	47.53 ± 14.6	36.66 ± 24.9	0.2320	NS
Increase in current, ΔI_{SC} (µA/cm^2)	29.78 ± 11.5	19.30 ± 9.69	0.0367	*
Time taken to reach peak (s)	95.40 ± 14.2	88.20 ± 8.70	0.1892	NS

Supplementary Table 5: Analysis of Hu, VIP, and ChAT-immunostained submucosal neurons.

Submucosal Hu⁺ neurons				
Parameters	WT (mean ± SD)	NL3 (mean ± SD)	p-value	Significance
Hu neurons per ganglion	8.500 ± 1.04	8.900 ± 1.26	0.6273	NS
Submucosal VIP⁺ neurons				
VIP neurons per ganglion	4.200 ± 0.963	4.620 ± 1.61	0.6611	NS
VIP neurons per ganglion (%)	57.64 ± 4.77	52.31 ± 15.5	0.5337	NS
Submucosal ChAT⁺ neurons				
ChAT neurons per ganglion	0.2750 ± 0.150	0.6000 ± 0.442	0.2064	NS
ChAT neurons per ganglion (%)	3.160 ± 3.04	6.568 ± 6.31	0.3589	NS

Supplementary Table 6: Analysis of caecal patch count, CD11c$^+$ cell morphology and Iba-1$^+$ cell morphology.

Caecal patch count					
Parameters	WT (mean ± SD)	NL3 (mean ± SD)		p-value	Significance
Caecal patch count (total)	1.000 ± 0	1.438 ± 0.629		0.0240	*
Caecal patch count (Main)	1.067 ± 0.258	1.000 ± 0		0.8994	NS
Caecal patch count (ILF)	0.2000 ± 0.561	0.467 ± 0.640		0.2008	NS
Caecal patch CD11c$^+$ cell morphology					
		NL3 (Main) (mean ± SD)	NL3 (ILF) (mean ± SD)		
Area (μm^2)	592.8 ± 194	543.6 ± 118	653.7 ± 146	0.5521	NS
Sphericity	0.6269 ± 0.0368	0.6329 ± 0.0512	0.5932 ± 0.0334	0.2962	NS
Volume (μm^3)	658.3 ± 281	584.7 ± 121	687.6 ± 173	0.7173	NS
Myenteric plexus Iba-1$^+$ cell morphology					
Number of Iba-1$^+$ cells	111.2 ± 16.8	109.2 ± 34.9		0.9108	NS
Area (μm^2)	2856 ± 665	2895 ± 232		0.9051	NS
Sphericity	0.4579 ± 0.0522	0.4792 ± 0.0184		0.4147	NS
Volume (μm^3)	4257 ± 1151	4694 ± 498.4		0.4575	NS
